# Supplementary material for: Molecular epidemiology and long-term survival analysis of HIV-1/AIDS patients infected with CRF07_BC, CRF01_AE and subtype B in Taiwan
Source: PLoS One. 2025 Jun 3;20(6):e0323250. doi: 10.1371/journal.pone.0323250 (PMC12132928; doi:10.1371/journal.pone.0323250)
Supplement: S1 Table — (DOCX) [file pone.0323250.s001.docx]

**Supplementary table 1.** Comparison of the mean CD4 counts of the first and last tests between HIV-1 subtype B and CRF07_BC patients who did not receive ART in Taiwan

| Subtype Cohorts |  | |  | |  |
| --- | --- | --- | --- | --- | --- |
|  | Subtype B | (CRF)07_BC | |  | |
| Variable | N=30 | N=178 | | *P* value | |
| **Mean CD4 counts of the first test,**  **(**± **/ standard deviation, WBC/mm3)** | 509.37  (±159.82) | 487.04  (±196.28) | | 0.556 | |
| **Groups of CD4 counts of the first test, N (%)** |  |  | |  | |
| <200 | 0 (0.00) | 6 (3.37) | | 0.696 | |
| 200-350 | 5 (16.67) | 41 (23.03) | |  | |
| 351-499 | 10 (33.33) | 58 (32.58) | |  | |
| >=500 | 15 (50.00) | 72 (40.45) | |  | |
| NA* | 0 (0.00) | 1 (0.56) | |  | |
| **Mean CD4 counts of the last test,**  **(**± **/ standard deviation, WBC/mm3)** | 494.31  (±239.42) | 476.65  (±229.4) | | 0.709 | |
| **CD4, N (%)** |  |  | |  | |
| Unchanged | 15 (50.00) | 84 (47.19) | | 0.905 | |
| increase | 6 (20.00) | 29 (16.29) | |  | |
| decrease | 7 (23.33) | 50 (28.09) | |  | |
| NA | 2 (6.67) | 15 (8.43) | |  | |
|  |  |  | |  | |
| **The mean first viral load**, (± / standard deviation, copies/ml) | 56,658.7(±11,275.5) | 31,298.1(±3706.5) | | 0.359 | |

NA: not available.
