# Supplementary material for: Molecular epidemiology and long-term survival analysis of HIV-1/AIDS patients infected with CRF07_BC, CRF01_AE and subtype B in Taiwan
Source: PLoS One. 2025 Jun 3;20(6):e0323250. doi: 10.1371/journal.pone.0323250 (PMC12132928; doi:10.1371/journal.pone.0323250)
Supplement: Attachment — Submitted filename: Latter to Editor-revision-12-3_Final.docx [file pone.0323250.s004.docx]

November 29, 2024

Dear Editor:

Thank you for your messages and the reviewer’s comments. We found the reviewer’s comments insightful and very helpful. We are pleased and encouraged by the reviewer’s general comments to our manuscript-“the strength of the study is in the large number of patients it looks at that have prospective data” and “the possibility of comparing subtype characteristic and clinical outcomes in the same geographic location is a rare opportunity to study non-B subtypes which are not well represented in literature”. As requested by the reviewer, we have further enhanced and double-checked the statistical analysis, with the help of two scholars who were added in the authorship. Attached please find our revised article entitled “Molecular Epidemiology and Long-term Survival Analysis of HIV-1/AIDS Patients Infected with CRF07_BC, CRF01_AE and Subtype B in Taiwan” and the following point-by-point reply to the reviewer #1.

Reviewer #1: This is an important paper that looks at difference in clinical outcomes among different HIV subtypes in Taiwan. The strength of the study is in the large number of patients it looks at that have prospective data. One major confounder is that CRF07_BC is more common among PWID while subtype B and CRF01_AE are more common in among those who acquired HIV through sexual contact. Nevertheless, the possibility of comparing subtype characteristic and clinical outcomes in the same geographic location is a rare opportunity to study non-B subtypes which are not well represented in literature. I do have a few concerns about the finding and the assumptions the authors have made:

1. The statement in the introduction "...since HIV-1 viral load correlates strongly with AIDS disease progression (lines 97 to 98)" is not supported by any citation, and is not consistent with published literature as shown for instance in Rodríguez et al. Predictive Value of Plasma HIV RNA Level on Rate of CD4 T-Cell Decline in Untreated HIV Infection. JAMA. 2006;296(12):1498–1506.doi:10.1001/jama.296.12.1498 (https://jamanetwork.com/journals/jama/fullarticle/203435). If there is newer evidence supporting the authors' assertion, this should be cited.

1. A: Thank you for your comments. We have modified the description in the introduction (Since the extent of viremia, measured as HIV-1 RNA viral load, has been postulated to be the best available surrogate marker of HIV-1 disease progression^11^, we hypothesized that patients infected with CRF07_BC might have better survival than patients infected with subtype B if they have similar risk of transmission (lines 96-100) and add a reference (No. 11: J W Mellors 1, C R Rinaldo Jr, P Gupta, R M White, J A Todd, L A Kingsley. Prognosis in HIV-1 infection predicted by the quantity of virus in plasma. Comment Science. 1996 May 24;272(5265):1167-70.).

2. The methodology for determining subtypes is not nucleic acid sequence-based and uses an old method based on PCR product lengths. This can be problematic in an epidemics with multiple subtypes, as it is likely to misclassify recombinants between circulating subtypes depending on the recombination sites. Is it possible to sequence representative samples from the biobanks to verify that the subtyping is accurate?

A: Thank you for your comments. We not only used nested multiplex PCR, but also employed nucleic acid sequencing with phylogenetic tree analysis of representative samples to determine the genotypes of our patients. We have clarified this issue and add two sentences in the materials and methods (lines 125-126; lines 133-134).

3-1. Line 131 to 133 in the methodology states, "Once multiplex PCR showed two or more HIV-1 subtypes suggesting dual infection, serial PCR using a single pair of subtype-specific primers was used to confirm dual infection." I have looked through the results and I have not found any statement on whether dual infections were detected, and what significance these had on the findings. Moreover, finding two or more subtypes on the PCR products may be because of recombinants between circulating subtypes and not necessarily dual infection. Were steps made to differentiate this?

A: Thank you for your enquiry. Yes, we did find two patients who were MSM have dual infection with Subtype B and CRF01_AE. These two patients were excluded from our survival analysis to avoid confusion. Since we did not include these two patients for survival analysis, we did not conduct long-distance PCR combined with cloning and sequencing to differentiate the situation.

3-2. The higher death rate for CRF07_BC versus subtype B seems to be more of a function of the mode of transmission (PWID vs, MSM) as well as lower ART uptake than actual subtype differences. Was an attempt made to correct for these confounders through multivariate analysis and modelling to see if HIV subtype had a significant contribution to survival?

A: We fully agreed with your comments. We took another approach to correct these confounders through comparing the survival between patients infected with different subtypes in the same risk group. As shown in Figure 2F, we found that among IDUs, patients infected with CRF07_BC had better survival rate (74.3%) than patients infected with subtype B (45.7%) and the difference was statistically significant. These findings suggest that the transmission route is one major factor influencing death rate. By eliminating this confounder and comparing only patients with the same transmission route, we can more accurately assess the true impact of subtypes on survival outcomes. We have added these sentences in the discussion of the manuscript (lines 363-368).

4. Lines 229 to 236, please generate hazard ratios with one of the subtypes as the reference and include 95% confidence intervals to see if the survival rates are significant or if there is overlap.

The low number of CRF01_AE samples means there will be a wide confidence interval and the seemingly low survival in non-ART treated patients may not be significant. Having said that, there is literature that suggests that CRF01_AE is associated with faster disease progression and higher viral loads so this may be a real subtype-related effect.

A: Thanks for the comments. We have generated hazard ratios with 95% confidence intervals and added a new Table (Table 7). The results show the hazard ratio for the 16-year death rate being 3.84 (95% Confidence Interval: 2.68-5.49) for CRF07_BC and 5.43 (95% Confidence Interval: 2.78-10.64) for CRF01_AE, compared with subtype B. (lines 247-248).

5. Lines 238 to 243, please show hazard ratios and 95% confidence intervals (and p values if preferred) instead of stating the differences were "statistically significant."

A: Our permission of using the database from the National Health Insurance Database had expired. Therefore, we no longer can access the raw data for the analysis suggested by the reviewer. However, the log-rank test results show that the death rates among the three main subtypes of HIV-1 in the intravenous drug user population in Taiwan are significantly different.

6. Lines 289-290 "Interesting (sic), if we don’t consider the risk factors of transmission routes first, we found that overall,
CRF07_BC has a significantly higher death rate (8.2% vs. 22.8%) compared to subtype B patients.

Please fix this line since the grammar is wrong (use Interestingly), and the death rates stated are confusing - is 8.2% for CRF07 or B? If you mean for CRF07, then it should be (22.8% vs. 8.2%) so as to make the order of statement of subtypes more consistent. Moreover, it is clear that more B than CRF07 were on ART so this should be considered as well. A proper multivariate analysis and specific univariate analyses will clarify the strengths of these associations.

A: Thanks for your suggestion, we have corrected our statement in the manuscript (lines 294-295). Unfortunately, due to the reason mentioned above, we are no longer able to access the raw data to conduct the statistical analysis.

7. Line 319 to 332, the conclusions are not tight and coherent. Please indicate the mean or (better) median viral loads and 95% confidence intervals for each subtype in the text when stating that CRF07 has a lower viral load than subtype B. If you look at Supplemental Table 1, the p value for comparisons of mean viral loads is not significant (p=0.359). Given the wide nature of viral loads, using median count, a log transformation or binary conditions (less than 100,000 copies versus >100,000 copies) may show more significant differences.

A: Thanks for your suggestion. Based on our current data and your suggestions, we have modified the sentences in the discussion (lines 324 to 325).

8. The conclusion of higher death rates (lines 289 to 290) for CRF07 is not consistent with the conclusion of slower disease progression due to lower viral loads (lines 320 to 322) and better survival. This needs to be resolved.

A: Thank you for your comments. We have revised several sentences to make the conclusion clearer (lines 363-368).

9. There are many grammatical and typographical errors throughout the manuscript. It is recommended that the revised manuscript be subjected to professional copyediting before resubmission.

A. Thanks for your suggestions. We have hired professional editor to correct our manuscript.
