# Supplementary material for: Molecular epidemiology and long-term survival analysis of HIV-1/AIDS patients infected with CRF07_BC, CRF01_AE and subtype B in Taiwan
Source: PLoS One. 2025 Jun 3;20(6):e0323250. doi: 10.1371/journal.pone.0323250 (PMC12132928; doi:10.1371/journal.pone.0323250)
Supplement: Attachment — Submitted filename: Letter to Editor-revision-3-6-25_AC & PC.docx [file pone.0323250.s005.docx]

March 6, 2025

Dear Editor:

Thank you for your message and the reviewer’s comments. We appreciate reviewer #1’s favorable review and recommendation for publication. We also found the reviewer #2’s meticulous comments insightful and helpful. As requested by the reviewer, we have further revised our manuscript accordingly. Attached please find our revised article entitled “Molecular Epidemiology and Long-term Survival Analysis of HIV-1 and AIDS Patients Infected with CRF07_BC, CRF01_AE and Subtype B in Taiwan” and the following point-by-point reply to the reviewer #2.

Reviewer #2: The paper presented by Jen et al. presents a unique opportunity to look at differences in clinical outcomes among HIV-1 epidemics in the one distinct geographical location. Particularly noteworthy is the inclusion and number of samples from a variety of non-subtype B HIV-1 samples; the authors should be commended for this focus.

However, I have major concerns regarding the statistics and analysis performed on these samples, primarily in regard to drawing any conclusions based on long-term survival without considering and/or stratifying for antiretroviral therapy (ART) usage and adherence. ……

As such, I would suggest the following major revisions:

1. The populations observed in the two epidemics (B and CRF07_BC) are very different, as noted in lines 178-185. Each epidemic has been found to have variations in subtype, route of transmission and ART uptake, and likely has different underlying nuances based on differences in demographics. I would use a mixed effect model or the like for this analysis, as the viral subtype is not independent of other characteristics that may impact long term survival. While I believe there has been some attempt to do this, particularly in figure 2, this approach to the data needs to be carried on throughout the entire analysis to ensure that the conclusions drawn are valid.

Answer: We appreciate the reviewer’s thoughtful comment and agree that a mixed-effects model would be a valuable approach to analyze clustered data like ours. However, we regret to inform the reviewer that we no longer have access to the raw data required for such an analysis. Our permission to use the National Health Insurance Database has unfortunately expired, and as a result, we are unable to perform the suggested mixed-effects model analysis currently. We recognize the importance of this methodology and would certainly consider it in future studies, should access to the data be renewed. However, the conclusion we made in lines 335-338 (as presented below) suggests that by focusing on a specific risk group, with all subjects under similar environmental conditions—such as lifestyle and socioeconomic status—the true effect of viral subtypes on survival rates could be investigated more independently.

“Moreover, among the PWID who had been genotyped, CRF07_BC patients had better survival rate than patients infected with subtype B or CRF01_AE and the differences are statistically significant (Fig. 2F). These findings support our hypothesis that under the premise of same risk factor, a lower viral load in CRF07_BC patients can contribute to their slower disease progression.”

2. Given the association with CRF07_BC with people who inject drugs in this epidemic, is there also an association in not just uptake, but also adherence to ART (i.e. achieving adequate viral suppression) for this subtype? This may also lead to differences in long-term survival. I do understand from the discussion (lines 357 – 363) that looking granularly at this may not be possible, however it does remain a significant issue that should be discussed.

Lines 200-211: individuals from the subtype B epidemic were found to have a better treatment response to ART through monitoring CD4 levels over time. However, was this analysis limited to only individuals adhering to therapy? Again, while I appreciate this may not be possible, I believe this needs to be made more explicit as adherence may be a significant factor here, particularly among people who inject drugs.

Table 4 and lines 214-226, the data does not seem to be stratified for ART usage. This is a major confounder of the results, given that ART significantly increases lifespan, something the authors also find in lines 241-242. This may be influencing the results presented here, as those individuals with a lower death rate also have the highest uptake of ART, though this is not taken into account.

Lines 229-237, again, the data does not seem to be stratified for ART usage, even though a significant association between the different subtypes and ART uptake has been shown prior (lines 188-192).

Answer: We appreciate the reviewer’s comment. We agree that adherence to ART is an important factor for long-term survival. Unfortunately, again, we no longer have access to the raw data required for such an analysis. Our permission to use the National Health Insurance Database has unfortunately expired, and as a result, we are unable to perform the analyses suggested by the reviewer. However, to clearly notify readers of this major point, we added “Assuming these individuals stringently adhere to their prescribed ART,” (Line 202-03) in the Results section, and “Lastly, given the adherence to ART is a known determinant for HIV-1 treatment outcome, particularly in IDUs, such information were unfortunately unavailable for our patients.”(Lines 365-67) in the Discussion section.

3. I also have the following suggested minor revisions:

3-1. Lines 69-70: ART is the key to controlling the epidemic; it is not a question of understanding (“being baffled”), but rather an issue of access to therapy.

Answer: We appreciate the reviewer’s comment. We have changed our sentence to “disease control of human immunodeficiency virus type 1 (HIV-1) remains manifestly inadequate.” (Line 70)

3-2 Lines 132-141: it is not immediately clear when the genotyping was performed, though I’m assuming from Figure 1 a blood draw was taken at/near the time of diagnosis. It may be helpful to make this explicit. While also unusual to not perform genotyping analysis by sequencing all samples (rather only a subset were sequenced), I do appreciate this is a historical data.

Answer: We appreciate the reviewer’s comment. To clarify the timing of blood draw and genotyping, we have modified our sentence to “blood samples from participants were collected (at the time of diagnosis).” (Line 134)

- Lines 205-206: long term non-progressors generally refers to individuals who are not taking ART, however I believe this paragraph/Table 3 only refers to individuals who are taking therapy.

Answer: We appreciate the reviewer’s comment. To clarify, we have modified our sentence to “for subtype B patients who received ART increased 13.8% (from 55.5% to 69.3%).” (Line 208)

4. Finally, though I appreciate it can be difficult, I would also encourage the authors to consider using person-centered language throughout the manuscript (e.g. people who inject drugs or people living with HIV) as per the UNAIDS terminology guidelines ( <https://www.unaids.org/en/resources/documents/2024/terminology_guidelines>)

Answer: We appreciate the reviewer’s comment. We agree the importance of using person-centered language. We have adapted person-centered language and modified the entire manuscript as per the UNAIDS terminology guidelines, which was cited in the acknowledgement. (Line 387-88)
